# The Phenotype of Hormone-Related Allergic and Autoimmune Diseases in the Skin: Annular Lesions That Lateralize

**DOI:** 10.1155/2012/604854

**Published:** 2012-12-17

**Authors:** Ramya Kollipara, Chetna Arora, Colleen Reisz

**Affiliations:** School of Medicine, University of Missouri-Kansas City, 2411 Holmes Street, Kansas City, MO 64108, USA

## Abstract

*Introduction*. Sexual dimorphism with an increased prevalence in women has long been observed in various autoimmune, allergic, and skin diseases. Recent research has attempted to correlate this female predilection to physiologic changes seen in the menstrual cycle in order to more effectively diagnose and treat these diseases. *Cases*. We present five cases of cutaneous diseases in women with annular morphology and distributive features that favor one side over the other. In all cases, skin disease improved with ovarian suppression. *Conclusion*. Sexual dimorphism in the innate and adaptive immune systems has long been observed, with females demonstrating a more vigorous immune response compared to males. Female sex hormones promote T and B lymphocyte autoreactivity and favor the humoral arm of adaptive immunity. In addition to ovarian steroidogenesis and immunity, intricate pathways coexist in order to engage a single oocyte in each cycle, while simultaneously sustaining the ovarian reserve. Vigorous proinflammatory, vasoactive, and pigment-related cytokines emerge during the demise of the corpus luteum, influencing peripherical sex hormone metabolism of the level of the macrophage and fibroblast. We propose that annular and lateralizing lesions are important manifestations of hormone-related inflammation and recognition of this linkage can lead to improved immune and reproductive health.

## 1. Introduction

Gender is an important variable in many diseases [1]. Women with asthma are at higher risk for developing severe disease in adulthood by a ratio of 2 : 1. Gender differences also exist in lupus, and other autoimmune diseases [2]. Similarly, there are gender differences in skin diseases such as granuloma annulare, palmoplantar pustulosis, and morphea. Attempts to explain these differences have focused on the variable physiologic changes seen in the menstrual cycle. Recent updates in fertility-based medicine have dramatically increased our understanding of the recruitment and development of a dominant follicle. Many of the events in the ovary provoke inflammatory cytokines and antibody formation that can affect organ systems unrelated to fertility.

**Figure 1:**
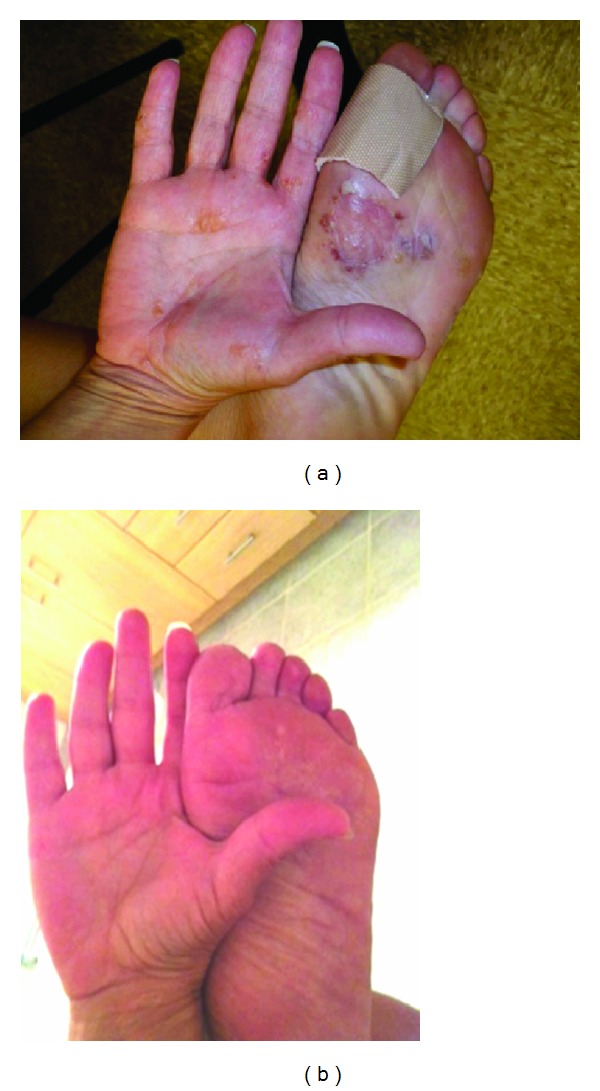
(a) 40-year-old female with abrupt onset of palmoplantar pustulosis. (b) Significant improvement occurred after three months of therapy with norethindrone-based oral contraceptive pills.

**Figure 2:**
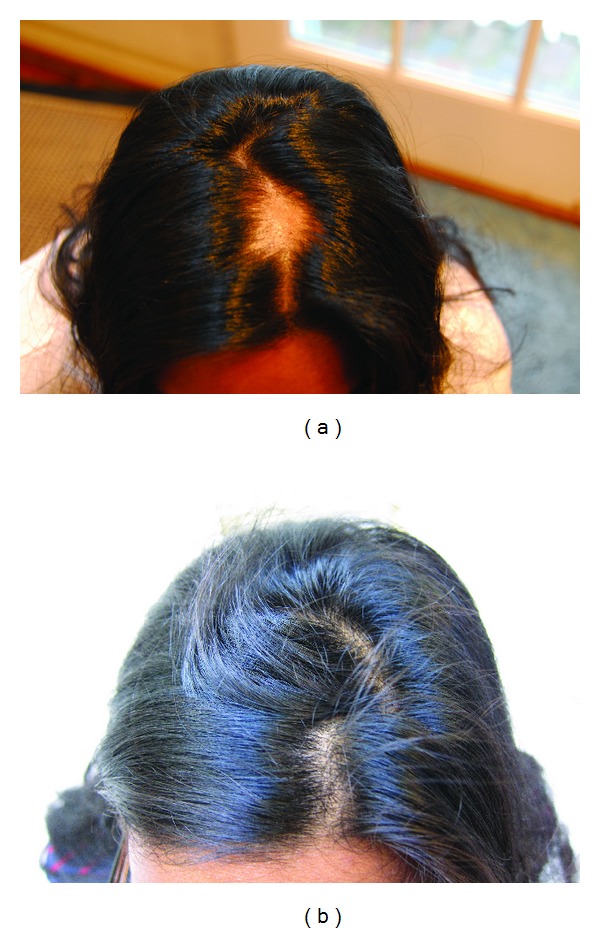
(a) A 23-year-old Indian female presented with a single patch of alopecia areata. (b) Hair regrowth is evident after 3 months of therapy with norethindrone and metformin.

**Figure 3:**
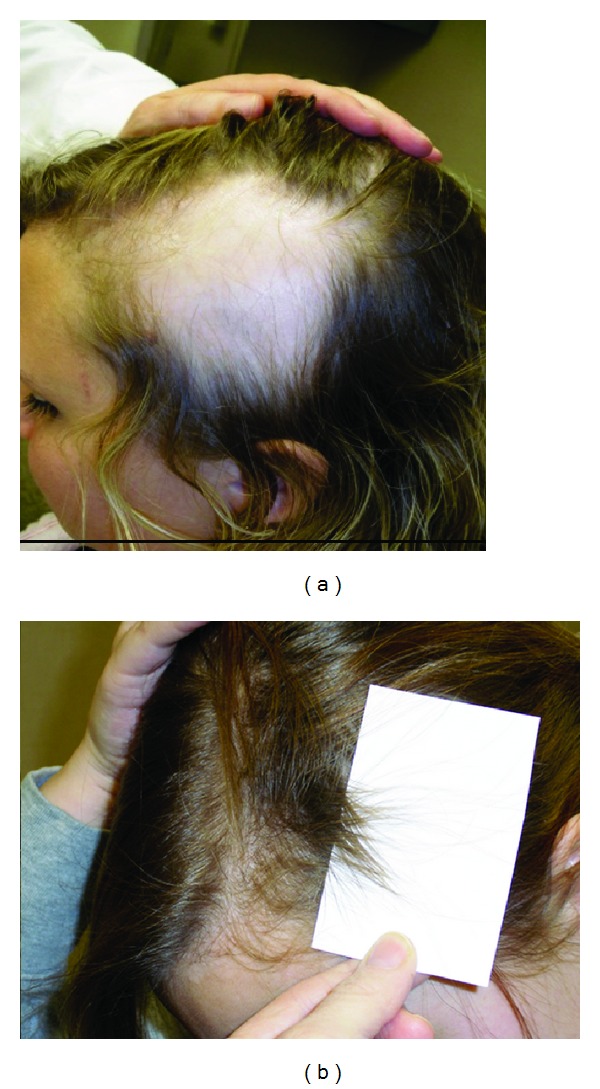
(a) A 19-year-old Caucasian female presented with a large temporal patch of alopecia areata. (b) Significant hair regrowth is evident after three months of therapy with norethindrone and metformin.

**Figure 4:**
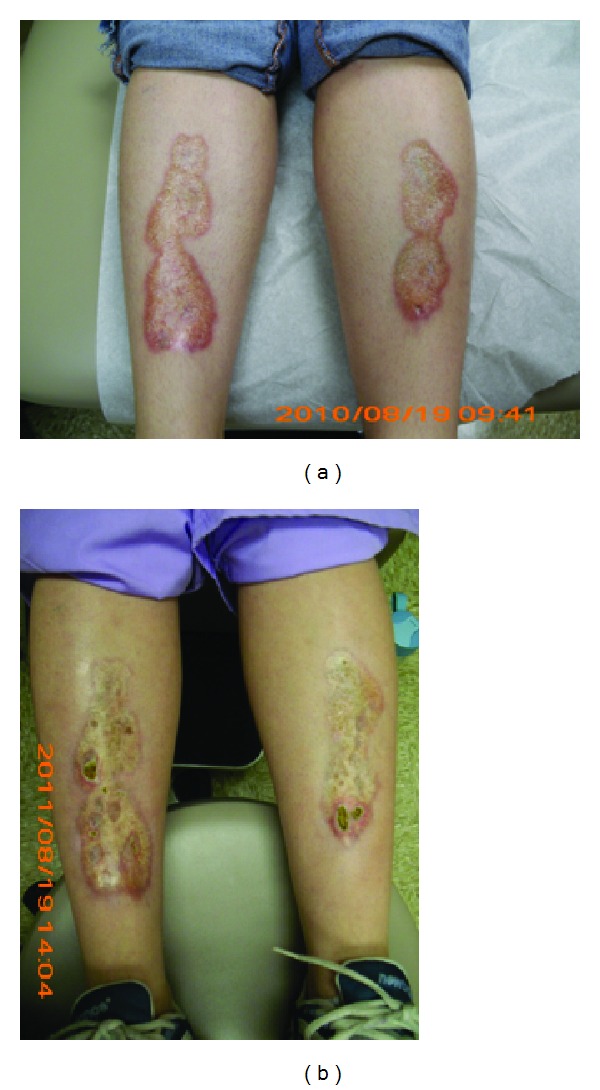
(a) A 25-year-old female with type I DM and onset of necrobiosis lipoidica at puberty. (b) A reduction in peripheral erythema and active border ensued after one year of therapy with norethindrone-based oral contraceptive pills.

**Figure 5:**
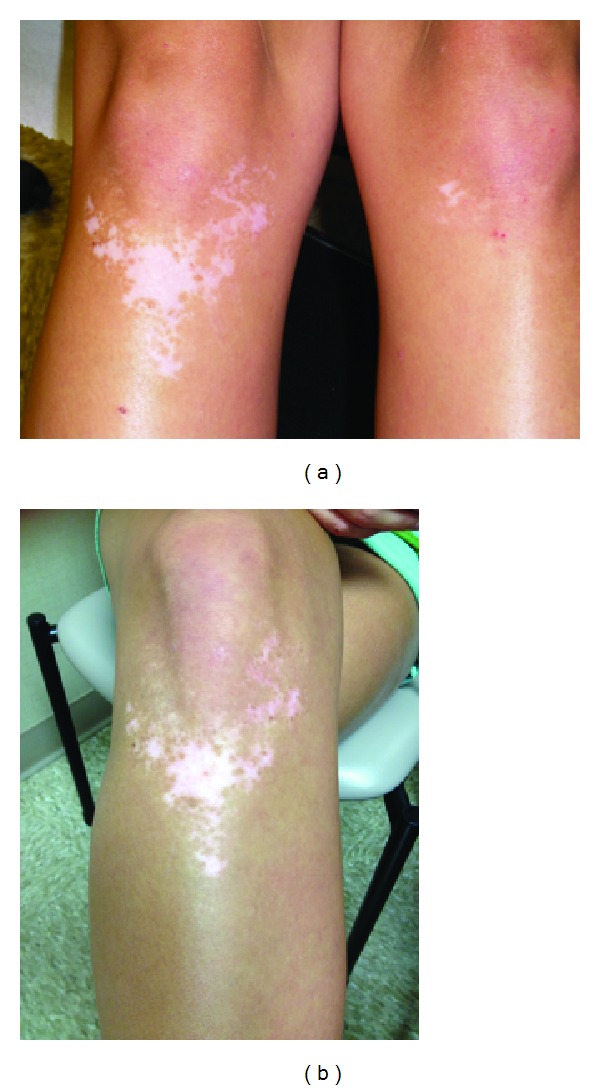
(a) A 20-year-old Caucasian female presented with vitiligo on the knees, with the right knee involved more than the left knee. (b) Repigmentation occurred after one year of norethindrone-based oral contraceptive therapy.

 Physicians taking care of women with allergic, autoimmune, and skin diseases need to recognize gender influenced systemwide inflammation. The following is a case series of women with skin diseases that improved with ovarian suppression. We review the stage-specific molecular changes in the ovary. The behavior of estrogen receptors in immune cells varies under inflammatory conditions and can dysregulate T-cell function, leading to B-cell hyperreactivity and an increase in autoantibody production per B cell. Immunomodulation is possible with progestins and metformin. The morphologic features shown below illustrate how annular lesions that favored one side directed our differential diagnosis, laboratory testing, and treatment plan (see Figures 1–5). 

## 2. Discussion

### 2.1. Hormonal Effects on Immune Function

 Sexual differences in the immune system have long been observed, with females generally demonstrating a more vigorous immune response than males [3]. Thus, although females mount a dynamic immune response to infections, they are a more prone to autoimmune diseases and some types of cancer. The female's immune response exhibits high macrophage and neutrophil activity with more efficient antigen presenting activity [4]. Females have a higher ratio of T cells to total lymphocytes [5]. Furthermore, the female adaptive immune system is primarily regulated by B cells and CD4 Th2 cells, while the male adaptive immune system is driven by CD8 and CD4 Th1 cells [6]. Females also have higher levels of immunoglobulins at baseline and when stimulated by infection or immunization [3]. Although many factors, including sex hormones, genetics, environmental factors, and microchimerism, are thought to influence this gender difference in immune response, the effect of sex hormones on immunity is the only one that has been widely studied [4].

Sex hormones, including estrogen, progesterone, and prolactin, affect immune cells via intracellular receptors. Intracellular estrogen receptors are found in B and T lymphocytes, neutrophils, macrophages, NK cells, bone marrow, and thymic stromal cells [7]. In mature lymphocytes, estrogen affects cellular activity by amplifying signal transduction [6]. Progesterone receptors are only found in lymphocytes of pregnant females as they are induced during pregnancy [3]. Prolactin receptors are found in B and T lymphocytes and stimulate gene transcription, T lymphocyte proliferation, and B lymphocyte antibody secretion [6]. 

 Ultimately, estrogen tends to favor Th2 cell line development by affecting the maturation and development of B and T lymphocytes [4]. Estrogen's influence on B-cell development favors B-cell autoreactivity and the production of immunoglobulin [8, 9]. In terms of T-cell development, estrogen decreases thymic T-cell production while promoting T-cell production in the liver. This allows autoreactive T lymphocytes to avoid tolerance induction and negative selection [8]. Estrogen also favors the development of the CD4 T cells over CD8 T cells and promotes the production of IL-4, IL-5, and IL-10 cytokines over other cytokines [4]. Inflammatory cytokines can also change the peripheral metabolism of estrogen by changing aromatase behavior, leading to androgen depletion and an overproduction of harmful estrogen metabolites [10].

The X chromosome contains many genes that affect immune function [11]. Approximately 10–15% of X linked genes escape silencing and are expressed from both x chromosomes, so physiologically some genes achieve double or null expression [1]. Differences in the expression of immune-related genes can cause a breakdown of the self-tolerance and persistence of autoreactive lymphocytes for X linked antigens.

### 2.2. The Physiology of the Ovarian Cycle

#### 2.2.1. Selecting the Dominant Follicle

 Physiologically, during each menstrual cycle, several follicles simultaneously mature in an attempt to reach ovulation. This cluster of maturation has been proposed to be secondary to rising levels of follicular-stimulating hormone (FSH). This follicular wave, stimulated by FSH, reciprocally causes a proportional decline depending on the number in the cohort stimulated and the amount of their released secretions. From there, only the dominant follicle (DF) escapes atresia when the FSH surge equally declines [12]. This follicle may have the dual benefit of having the lowest FSH requirement via enhanced responsiveness, as well as the ability to amplify its FSH-stimulated granulosa cells. This low requirement may also be due to increased, or maintained high FSH receptor mRNA expression with FSH binding, in turn allowing it to escape atresia and reach the >8 mm diameter threshold in becoming the DF. In addition, after the continued reduction of FSH, the DF must then gain FSH independence (and the concordant LH dependence) in order to sustain and differentiate [13].

#### 2.2.2. Maintaining the Follicular Pool

**Figure 6:**
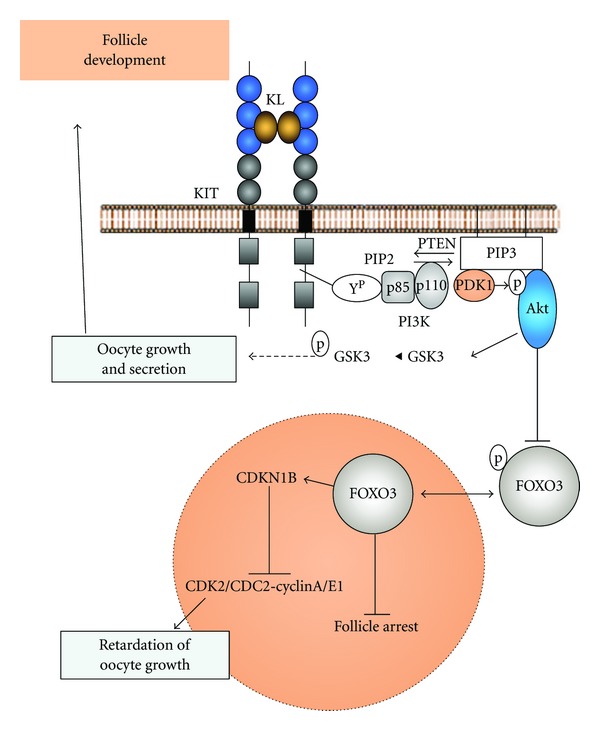
McLaughlin and McIver [14].

 The oocyte, itself, governs follicular activation, and within it, the PTEN pathway (phosphatase and tensin homolog deleted on chromosome 10) determines the actual initiation of growth, or the preservation of the dormant follicular pool. PTEN is part of a larger pathway and is a negative regulator of PI3K (phosphoinositide 3-kinase) signaling pathway. P13k, through the action of many downstream substrates (i.e., FOXO3), positively activates the primordial follicle. Thus, PTEN can altogether prevent this and maintain dormancy (Figure 1) [14]. In a study, PTEN was deleted from the oocytes of the primordial pool via oocyte-specific transgene expression, and as a result, global activation of the follicles occurred with the secondary depletion of the pool [15, 16]. This process is irreversible once begun, and all those not selected for continued development ultimately undergo atresia and subsequent premature ovarian failure (see Figure 6).

#### 2.2.3. Immune-Induced Premature Ovarian Failure

Several different causes of premature ovarian failure (POF) are implicated, ranging from iatrogenic (chemotherapy, radiation, and surgery) to autoimmune and genetic conditions. It is estimated that 10–20% of POF is caused by autoimmunity, yet this remains controversial as the exact correlation is still unclear [17]. The possible etiologies include circulating anti-ovarian antibodies, plasma and lymphocytic cell penetration of the ovary, adaptation of T-cell subsets, associations with other autoimmune disorders, and the recovery of ovarian function after the improvement of the baseline autoimmune disorder [18]. POF, in association with an amplified autoimmune reaction, can cause hastened atresia and blighted folliculogenesis. For example, patients with both POF and adrenal autoimmunity (i.e., Addison's disease) can have autoantibodies recognized by several types of steroid-producing cells within not just the adrenal cortex, but also the placenta and ovary—further demonstrating a shared autoimmune focus. Even in the patients with isolated or idiopathic POF, the level of these antibodies remains elevated [19]. Several potential markers can be used to assess autoimmunity, such as antibodies to the LH receptor, FSH receptor, and the zona pellucida [20]. General antiovarian antibodies represent an independent marker of autoimmune ovarian disease, yet this remains controversial in terms of its specificity. The level of this antibody seems to appear before the onset of symptoms, possibly functioning as a marker to follow the progression of POF [19]. Ultimately, the interplay between the upregulation and suppression of the primordial pool of follicles within the ovary is intricate and is maintained by external events as well as various molecules, including, but not limited to, PTEN, P13k, and antimullerian hormone. Loss of these would result in total activation and subsequent premature ovarian failure [21].

#### 2.2.4. Preparation and Secondary Ovulation

Once the dominant follicle has emerged and bypassed all the negative-regulating factors, multiple preparatory events, including vesiculation, mucification, and cumulus rupture, line up to take place. Prior to ovulation, as the secondary follicle matures, a fluid-filled cavity, or antrum, forms adjacent to the oocyte. This separates the granulosa cells surrounding the oocyte into two populations: cumulus cells that encapsulate the oocyte and the mural granulosa cells that form the follicular wall. Differentiation of this cell population and the formation of the antrum mark the transition of the secondary follicle to the antral (Graafian) or tertiary follicle [22]. In the mature antral follicle, the cumulus cells secrete hyaluronic acid, a type of glycosaminoglycan that binds to cumulus cells via linker proteins. As the hyaluronate is hydrated, the cumulus cells spread apart and become entrenched in a mucified extracellular matrix [22, 23]. This expands the volume of the cumulus-oophorus complex (COC) to almost 20–40 times its initial volume [24]. This process is called mucification [23]. As the COC further expands, it dissociates from the follicular wall. This is accompanied by the preovulatory LH surge, which stimulates prostaglandin, leukotriene, and matrix-binding protein secretion by the mural granulosa and theca cells. As a result, the permeability of the follicle-blood barrier increases, leading to increased antral fluid, rising intrafollicular pressure, rupture of the follicular wall, and successive extrusion of the COC [24].

#### 2.2.5. Multifactorial Demise of the Corpus Luteum

 In the absence of fertilization and resultant trophoblastic human chorionic gonadotropin (HCG) secretion, the corpus luteum undergoes luteolysis, a dynamic process resulting in structural and functional regression. And as a result, progesterone production ceases just prior to the collapse of the corpus luteum organ [25]. Despite many studies, apoptosis (type I programmed cell death) and autophagy (type II programmed cell death) are simply suggested as the main mechanisms, but the exact trigger for luteolysis and the mechanism of structural involution are not fully understood [26]. In conjunction with the molecularly derived demise of this organ, local regulation of angiogenesis and luteal blood flow seem to play a major role as well [25]. Vascular endothelial growth factor (VEGF) and basic fibroblast growth factor (bFGF) are expressed the highest in the early luteal period, concurrently increasing vascular permeability and thus progesterone secretion. Initially, a positive feedback loop is created in which VEGF stimulates prostaglandin F2-alpha (PGF2-alpha), which then further increases VEGF. As the late corpus luteum ensues, a decrease in the blood supply occurs secondary to an overriding PGF-2 suppression of VEGF, and ultimately luteolysis transpires through the vasoconstriction and disconnect of an appropriate blood supply [27].

### 2.3. The Molecular Basis of Laterality

Women are considered to be genetic mosaics due to lyonization, or random X-chromosome inactivation in somatic cells [28]. During embryogenesis, one X-chromosome, at random, expresses the X-inactive specific transcript (XIST) gene thereby silencing that chromosome [29]. As a result of this process, each female is a mosaic of two cell populations, one in which her paternal X-chromosome is inactive and the other in which her maternal X-chromosome is dormant [30]. Although this process is intended to be random, skewed X-inactivation due to chance, genetic modifiers, or a survival instinct to silence X-chromosome can confer an evolutionary disadvantage [29]. The X-chromosome contains many genes responsible for immune function and the 10–15% of the genes that escape silencing can lead to double or null expression. 

X-chromosome-associated mosaicism also plays a significant role in the distribution of cutaneous diseases. Perhaps the most famous example of genetically influenced skin patterning is Blaschko's lines. First described by Alfred Blaschko in 1901, alternating bands of diseased and normal skin occur and are pathognomonically associated with over a dozen of X-linked skin disorders such as incontinentia pigmenti, anhidrotic ectodermal dysplasia, and hypomelanosis of Ito [28, 29]. 

Another form of patterning that occurs in cutaneous lesions is lateralization. This pattern is exclusively seen in X-linked congenital hemidysplasia with ichthyosiform erythroderma and limb defects (CHILD syndrome). This syndrome presents as scaly erythematous plaques on one-half of the body with abnormalities in ipsilateral internal organs and bones. This unique distribution is attributed to lyonization and a gene defect in the sonic hedgehog pathway that controls left-right patterning [30].

## 3. Conclusion

The influence of hormones on immunity has been the subject of recent reviews. Our goal was to align certain morphologic and distributive features in the skin with the variable physiology of the menstrual cycle. The ovary is pivotal in maintaining the hormonal equilibrium required for follicular development, preservation of the reproductive tract, development of secondary sexual characteristics, and general metabolic finesse [21]. 

 Progestins and androgens have immunomodulatory effects [31]. Synthetic progestins, in particular, can be used to suppress harmful communication between hormones and immune cells. The recent discovery of increased HIV transmission in women who use depot medroxyprogesterone (DMPA) has drawn attention to the untoward effects of reducing antiviral cytokines seen with this form of contraception. Other progestins have differing effects on cytokines than DMPA and may be beneficial in clinical situations that suggest allergy or autoimmunity. Norethindrone would be a good choice if ovarian and inflammatory suppression is the primary goal. 

 Other medications that impact hormone driven immunity include gonadotropin hormone releasing agonists and metformin. Metformin inhibits PTEN and preserves the dormancy of the follicular pool. This effect decreases the follicular mass, correcting the androgen and insulin discord seen in polycystic ovary syndrome [32]. Metformin also influences aromatase behavior, reducing the influence of inflammation on peripheral estrogen metabolism.
